# Antibiofilm Potential of Medicinal Plants against *Candida* spp. Oral Biofilms: A Review

**DOI:** 10.3390/antibiotics10091142

**Published:** 2021-09-21

**Authors:** Rafaela Guimarães, Catarina Milho, Ângela Liberal, Jani Silva, Carmélia Fonseca, Ana Barbosa, Isabel C. F. R. Ferreira, Maria José Alves, Lillian Barros

**Affiliations:** 1 AquaValor–Centro de Valorização e Transferência de Tecnologia da Água–Associação, Rua Dr. Júlio Martins n.º 1, 5400-342 Chaves, Portugal; rafaela.guimaraes@aquavalor.pt (R.G.); catarina.milho@aquavalor.pt (C.M.); jani.silva@aquavalor.pt (J.S.); 2 Centro de Investigação de Montanha (CIMO), Instituto Politécnico de Bragança, Campus de Santa Apolónia, 5300-253 Bragança, Portugal; angela.liberal@ipb.pt (Â.L.); iferreira@ipb.pt (I.C.F.R.F.); 3 Instituto Politécnico de Bragança, Escola Superior de Saúde (ESSa), Campus de Santa Apolónia, 5300-253 Bragança, Portugal; carmeliaamaralfonseca@hotmail.com (C.F.); pbarbosatb@gmail.com (A.B.)

**Keywords:** *Candida* spp., oral disease, oral biofilm, infections, medicinal plants, plant extracts, natural compounds, antibiofilm strategies

## Abstract

The use of natural products to promote health is as old as human civilization. In recent years, the perception of natural products derived from plants as abundant sources of biologically active compounds has driven their exploitation towards the search for new chemical products that can lead to further pharmaceutical formulations. *Candida* fungi, being opportunistic pathogens, increase their virulence by acquiring resistance to conventional antimicrobials, triggering diseases, especially in immunosuppressed hosts. They are also pointed to as the main pathogens responsible for most fungal infections of the oral cavity. This increased resistance to conventional synthetic antimicrobials has driven the search for new molecules present in plant extracts, which have been widely explored as alternative agents in the prevention and treatment of infections. This review aims to provide a critical view and scope of the in vitro antimicrobial and antibiofilm activity of several medicinal plants, revealing species with inhibition/reduction effects on the biofilm formed by *Candida* spp. in the oral cavity. The most promising plant extracts in fighting oral biofilm, given their high capacity to reduce it to low concentrations were the essential oils extracted from *Allium sativum* L., *Cinnamomum zeylanicum* Blume. and *Cymbopogon citratus* (DC) Stapf.

## 1. Introduction

Medicinal plants have been used for several centuries to treat a wide variety of ailments. In recent years, the investigation into molecules derived from these plants, which play a fundamental role in the resistance of various pathogens, has boosted the study of their antibacterial and/or antibiofilm properties [1,2,3]. Some plant compounds can interact with bacterial proteins and cell membrane structures, damaging them and reducing their fluidity, while inhibiting their nucleic acid synthesis and interfering with the energy metabolism of the microorganisms themselves [2,4,5]. Additionally, the study of the antibiofilm properties associated with these molecules has revealed that, in addition to their fungicidal/bactericidal effect, other underlying mechanisms can lead to biofilm suppression, namely, disturbances at the level of bacterial regulation mechanisms [6].

The biofilm is a more resistant form of microbial existence on solid surfaces and air–liquid interfaces in which microorganisms multiply in a matrix of self-produced extracellular polymeric substances (EPS) [7]. Its resistance is directly related to the natural survival characteristics of the microbial cells that live in these communities. The slower growth of cells associated with the biofilm, as opposed to free-living microbial cells, and the tight regulation of the cellular processes, stand out, and are mainly caused by the more restricted contact of the cells inside the biofilm with external nutrients. In addition, the presence of an EPS matrix that hinders the action of antimicrobials contributes even more to the resistance of biofilms, since this matrix acts as a diffusion barrier against small molecules [8,9].

Biofilms can be found in a variety of surfaces, both biotic and abiotic. Particularly in the oral cavity, biofilm can be found in the teeth and mucosal surfaces and are thought to consist of approximately 700 bacterial species, 100 fungal species, and some viruses [10]. Since these microorganisms coexist in the same environment, there is the possibility of interactions between different species, a factor that can make an oral infection more difficult to treat, creating an environment of protection and tolerance for microorganisms against conventional antimicrobial agents [11].

One of the main groups of microorganisms that can be found in the normal oral flora is the genus *Candida*, which is composed of dimorphic commensal yeast. Although *Candida* species are mainly nonpathogenic, when an imbalance in the oral microbiome occurs, they are the main pathogens responsible for the occurrence of fungal infections in the oral cavity [12]. One of the key virulence factors associated with these microorganisms is their ability to adhere to oral surfaces and form biofilms, which function as a reservoir for this type of fungi, both in teeth and mucosal surfaces [13,14]. Several factors contribute to the unbalanced colonization and biofilm formation in the oral cavity by *Candida* spp., namely, low salivary flow, low pH and poor oral hygiene among others [15]. As an opportunistic pathogen, this yeast can also cause disease when the host’s immune system is debilitated by the appearance of pathologies such as diabetes mellitus and Human Immunodeficiency Virus (HIV) infection, and by the use of broad-spectrum antibiotics, among others [16]. Additionally, as they are one of the largest acid producers in the oral cavity, *Candida* fungi can also be at the origin of dental caries through a localized infectious process [17,18,19].

Once the establishment of pathogenic oral biofilms occurs, the risk of the occurrence of systemic infections increases, as does the resistance of these infections to conventional antimicrobial therapies [20]. Currently, the treatment of *Candida* infections in the oral cavity is mostly done using broad-spectrum antimicrobials, however, conventional biocidal agents can cause substantial side effects if administered in high concentrations, including vomiting, diarrhea, mucosal desquamation, tooth discoloration, etc. [11,19]. Given the harmful effects of traditional antimicrobial agents, and the increasing microbial resistance to them, natural plant products have been pointed out as a safe and efficient alternative for the treatment of *Candida* infections in the oral cavity since, together with their anti-inflammatory, antioxidant, and analgesic properties, they also exert antimicrobial and antibiofilm effects over *Candida* spp [21].

## 2. The Bioactive Compounds of Plants

Folk knowledge about the medicinal use of plants has been transmitted for centuries [22]. In recent years, much of the ethnopharmaceutical research has been focused on more specific approaches in order to evaluate and understand the biological and pharmaceutical effects of medicinal and aromatic plants [22]. Plants are rich in a wide variety of secondary metabolites which play an important role in the defense against numerous pathogens. These molecules are also involved in adaptation to biotic and abiotic stresses, protection against ultraviolet radiation, oxidation of molecules, nutritional and water stresses, while performing functions at the tissue level structure, being able to add flavor and color to plant products [23].

Presently, about 200,000 different plant secondary metabolites have been isolated and identified [24]. They can be classified based on their chemical structures and/or biosynthetic pathways [25]. A simple classification includes three main groups: terpenoids (polymeric isoprene derivatives and biosynthesized from acetate via the mevalonic acid pathway), phenolics (biosynthesized from shikimate pathways, containing one or more hydroxylated aromatic ring), and alkaloids (nonprotein nitrogen-containing compounds, biosynthesized from amino acids, such as tyrosine) [26]. Terpenoids, the condensation products of C5 isoprene units, are the main components of plant volatiles and essential oils [27]. They present many important properties, including anti-insect, antimicrobial, antiviral, and antiherbivore properties [28]. Phenolic compounds are widely found in fruits, seeds, leaves, roots, and stems, and are known for their strong antioxidant ability and their anticancer, anti-inflammatory, hypolipidemic, and hypoglycemic properties [29,30]. They have at least one aromatic ring with one or more hydroxyl groups attached, ranging from low molecular weight molecules to large and complex ones [31]. Alkaloids are usually cyclic organic compounds that contain at least one nitrogen atom in an amine-type structure [32]. These compounds are known to possess varied biological activities such as antimicrobial and antimalarial properties, among others [33].

**Figure 1:**
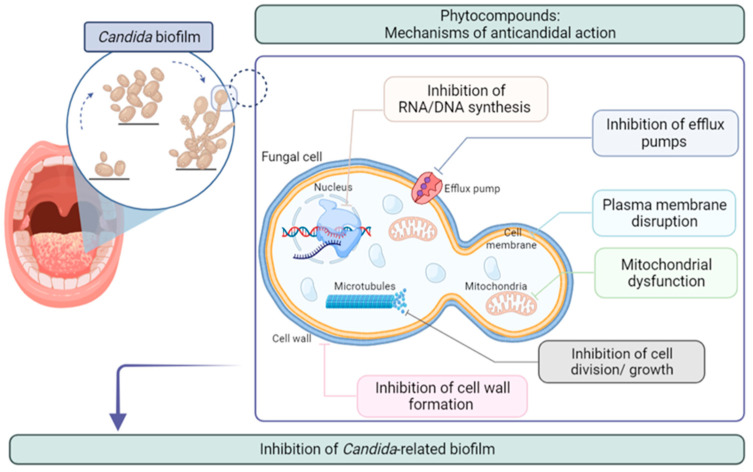
Mechanisms of action of phytocompounds against *Candida* spp. (Created with BioRender.com).

Many studies have been published regarding bioactive properties such as antioxidant [34,35], antitumoral [31,36], analgesic/anti-inflammatory [29,37], immunostimulant [38], antiseptic, and antimicrobial [39,40,41]. The antimicrobial and/or antibiofilm activity linked with some of these compounds is closely related to their ability to inhibit the synthesis of nucleic acids, disrupt the plasma membrane, inhibit efflux pumps, elicit mitochondrial disfunction, impair cell division and/or growth, and impair cell-wall formation, as shown in Figure 1 [42,43].

Given their strong bioactive potential, various types of phytocompounds are currently used in a wide range of fields such as food, pharmaceuticals, biomaterials, and environmental purification [44]. Regarding the ability of these compounds as antimicrobials, multiple studies have been conducted to determine their capability to fight oral infections caused by opportunistic pathogens such as *Candida* species [45,46,47,48]. The increased virulence of some *Candida* species such as *Candida albicans* is largely related to their ability to form biofilms which, as mentioned before, makes oral infections caused by these microorganisms very difficult to treat [49]. Taking this information into account, the use of plant-derived products to fight oral pathologies caused by *Candida* appears as an alternative to conventional antifungal therapy. In oral care, the use of natural products to prevent candidiasis is receiving much attention and many studies have reported the effects of medicinal plant extracts on the inhibition of oral pathogen growth and inhibition of surfaces adhesion to surfaces [50]. Some of the most prescribed antimycotic agents that are currently used target the synthesis of fungal cell membrane components that are not found in human cells, such as ergosterol [51]. However, there are few available antifungal compounds that show low levels of cytotoxicity, given the similarities between human and fungal cells, making it urgent to search for and identify new molecules capable of disrupting biofilms formed by *Candida* spp. and increase the arsenal of antifungal agents [52,53]. Knowing this, screening plants as potential sources of molecules with antifungal and/or antibiofilm properties can be considered an excellent approach to combat the formation of *Candida* spp. oral biofilms and the establishment of infections [54].

## 3. Opportunistic Fungal Infections Caused by *Candida* spp.

Currently, fungal infections affect millions of people every year, being the fourth leading cause of hematogenous infections worldwide. *Candida* spp., commensal microorganisms present in the normal microbial flora of the skin and mucosal surfaces (oral cavity, gastrointestinal tract, and vagina) of healthy individuals [55], are presented as the main responsible for the development of candidiasis, the most common invasive fungal disease in developed countries [56]. As commensals, *Candida* species are harmless; however, if the balance of normal flora is disrupted or immune defenses are compromised, these fungi can overrun the normal flora and cause disease. When the host’s immune status is impaired, two main types of *Candida* infection can be observed: superficial or invasive candidiasis. Superficial infections of the mucosal epithelial tissues are frequent in immunocompromised patients and include chronic atrophic stomatitis, chronic mucocutaneous candidiasis, and vulvovaginitis. In more severe cases, *Candida* species can enter the bloodstream (candidemia) and penetrate almost every organ in the body [57].

Seven *Candida* species are classified as clinically relevant, namely, *C. albicans*, *C. tropicalis*, *C. glabrata*, *C. parapsilosis*, *C. stellatoidea*, *C. kruseia*, and *C. kyfer*, with the species *C. albicans* being the most relevant since it is the most often isolated from deeper tissues, blood, and organs [58,59].

Candidiasis has been related, majorly, to *C. albicans* species, a dimorphic fungal organism that is normally present in the oral cavity in a nonpathogenic state but which, under propitious conditions, can transmute into pathogenic hyphae form due to changes in the normal conditions of the oral cavity, especially in patients with reduced immune function or in antibiotic treatment [60,61,62]. A variety of local and systemic predisposing factors can lead to the transition from commensal to pathogenic *Candida*, namely the use of dentures, corticosteroid inhalers, and xerostomia, and systemic factors such as immunosuppressive states, HIV infection, malnutrition, diabetes, systemic chemotherapy, and radiotherapy, among others [63]. Therefore, about 65% of oral candidiasis are identified in the elderly, usually due to the use of dentures, and other pathologies associated with this age group, and about 16.7% in patients with hematological disorders [64]. Other factors, such as the diversity of microorganisms, the presence of saliva, vascularization, contamination by food residues, and trauma resulting from lack of hygiene, increase the inflammatory process, healing time, and patient discomfort [65,66].

*Candida* spp. express a variety of virulence factors so that it can cause disease. Biofilm formation in *Candida* spp. and the transition from planktonic to sessile form are mainly associated with a high resistance to antimicrobials. Other mechanisms include the expression of resistance genes, particularly those encoding efflux pumps, and the presence of persistent cells [67]. The interaction of bacteria and *Candida* within the biofilm is increasingly evident, however, the role of fungi in the progression of inflammation and the prognosis of oral infections remains uncertain [68].

Currently, there are only four main classes of antifungals in clinical use: azoles, polyenes, echinocandins, and pyrimidine analogs. The lack of antifungal diversity dramatically decreases the chances of treatment success and increases the probabilities of a fatal outcome if the pathogen is resistant to one or more drugs [69]. Therefore, the search for alternative products and phytochemicals isolated from plants and used in traditional medicine is considered a good alternative to conventional synthetic drugs, offering a wide range of molecules with antimicrobial and/or antibiofilm properties to combat oral candidiasis.

## 4. Plant Extracts against Oral Biofilm Formed by *Candida* spp.

Most of the available antifungals are either ineffective against *Candida* biofilms or exhibit activity at very high concentrations [70]. Concerning microbial resistance, pharmacotherapy has reached its limit, threatening the effective prevention and treatment of an ever-increasing range of infections. These limitations have led to the search for novel molecules with antibiofilm potential. Plants are rich sources of bioactive molecules exhibiting various biological and pharmaceutical properties. Therefore, in recent years, new clinical approaches using natural phytocompounds have been the subject of several types of research, considering the composition of natural plant products in molecules with antimicrobial and/or antibiofilm potential. Table 1 presents some of the plant species whose extracts hold compounds with antifungal/antibiofilm activity against *Candida* spp. Moreover, extracts able to inhibit biofilm formation and/or eradication in more than 99%, at concentrations ≤ 1 mg∙mL^−1^, were chosen for discussion.

*Allium sativum* L. (Amaryllidaceae) is an aromatic herbaceous annual plant, one of the oldest authenticated and most important herbs that have been used since ancient times in traditional medicine. It is one of the most described plant species with proven antifungal, antimicrobial, anti-aging, as well as anticancer properties, which have been confirmed by epidemiological data from human clinical studies [71]. This specie and its active components have been also reported to reduce the risk of diabetes and cardiovascular diseases [72,73]. *A. sativum* antibiofilm properties against oral cavity yeast were studied by Fahim et al. [74] who demonstrated that, for a concentration of 8.00 µg∙mL^−1^, *A.* *sativum* L. essential oil presented > 99.9% of growth reduction on biofilm of *C. albicans* ATCC 14053. The ability of this essential oil to inhibit biofilm formation seems to be correlated with its phenolic profile, with allicin, alliin and ajoene being the major compounds found in it [75].

Essential oils from some plants have shown high antifungal and/or antibiofilm activity against *Candida* species. An example of this are the species of *Cinnamomum cassia* (L.) J. Presl, *Cinnamomum zeylanicum* Blume, *Cymbopogon citratus* (DC.) Stapf, *Cymbopogon nardus* L. Rendle, and *Cymbopogon winterianus* Jowitt.

*C. cassia* (L.) J.Presl (Lauraceae), also known as “Chinese cinnamon,” is a well-known aromatic plant that has been widely cultivated and utilized to treat diabetes, ovarian cysts, stomach spasms, kidney disorders, high blood pressure, and menstrual disorders [76], and presents antimicrobial, antioxidant and antifungal properties [77]. *C. zeylanicum* Blume (Lauraceae) is an ever-green perennial plant that is used as a culinary herb [78]. This species presents several pharmacological properties such as antimicrobial, antioxidant, antifungal, and anticancer [79]. When it comes to oral health, a study performed by Almeida et al. [80] demonstrated that *C. cassia* essential oil, at a concentration of 1.00 mg∙mL^−1^, exerts more than 99.9% reduction in oral biofilm formation caused by *C. albicans* ATCC 90028, while *C. zeylanicum*, at a concentration of 1.6 µg∙mL^−1^, leads to more than 99.75% reduction in oral biofilm formation caused by *C. albicans* ATCC 10231. The high percentage of biofilm reduction shown by these two plants is attributed to the major phytocompound found in both species, the cinnamaldehyde. Cinnamaldehyde is a phenylpropanoid that may act on the cell membrane, likely binding to enzymes involved in the formation of the cytoplasmic membrane in fungal cells [81].

*C. citratus* (DC.) Stapf (Poaceae), commonly known as lemongrass, is an aromatic plant widely distributed around the world. It is used as a food flavouring, and is commonly consumed in teas and soups, but it may also be served with poultry, fish, beef, and seafood. Lemongrass essential oil exhibits a number of biological activities, including antioxidant [82], anti-inflammatory [83], antimicrobial [84], antifungal, and antibiofilm properties [85]. Almeida et al. [80] used the essential oil from *C. citratus* as an antifungal agent against *C. albicans* ATCC 10231 biofilms, and reported that, at the concentration of 6.4 µg∙mL^−1^, this essential oil was able to reduce the number of viable cells present in the biofilm by 99.79%. In this case, citral and neral were two of the main compounds found, which are known to hold antifungal properties [86,87].

*C. nardus* L. (Poaceae), popularly known as citronella, is a grass cultivated in subtropical and tropical regions of Asia, Africa, and America, including Brazil [88], The essential oil extracted from its leaves is commonly used in perfumes, the production of cosmetics, and as an insect repellent. Several studies have demonstrated the antiviral [89], antibacterial [90], and antifungal activities [91] of this oil. *C. winterianus* Jowitt (Poaceae) is an important aromatic plant cultivated in India and Brazil. In folk medicine, it is used for the treatment of anxiety, as a sedative, and for pain disorders [92]. Some studies demonstrated that the plant has anticonvulsant effects [93], anti-larvicidal effects against *Aedes aegypti* [94], and antibacterial and antifungal effects, including anti-*Candida* action [95]. The essential oils extracted from *C. nardus* L. and *C. winterianus* Jowitt species showed, in different studies, to be highly effective in combating *C. albicans* oral biofilms. *C. nardus* showed, at a concentration of 32.0 µg∙mL^−1^, an adherence inhibition of *C. albicans* ATCC 76645 higher than 99.0%, [68] and the application of *C. winterianus* essential oil, at a concentration of 1.00 mg∙mL^−1^, led to a reduction of *C. albicans* ATCC 90028 oral biofilm formation by more than 99.0%. In both species, the authors attributed the antibiofilm potential to the main compound identified in these species, namely citronellal. Citronellal is known to affect *C. albicans* cell growth by interfering with cell-cycle progression through the arrest of cells in S phase and affecting membrane integrity [96].

*Solidago virgaurea* L. (Asteraceae), commonly known as goldenrod, is a medicinal plant that is common throughout the world. In the literature, this plant is described as possessing a variety of medicinal properties such as antioxidant, anti-inflammatory, analgesic, spasmolytic, antihypertensive, antibacterial, antifungal and antitumor, among others [97]. Chevalier et al. [98] evaluated the effect of the extracts from two *S. virgaurea* subspecies, *S. virgaurea* subsp. *alpestris* and *S. virgaurea* subsp. *virgaurea*, on *C. albicans* oral biofilm growth. The results obtained showed that, at an extract concentration of 250 µg∙mL^−1^, *S. virgaurea* subsp. *alpestris* inhibition of oral biofilms from *C. albicans* IM003 was higher than 99.5%, and that *S. virgaurea* subsp. *virgaurea* inhibited the oral biofilm formation by *C. albicans* IM001 by more than 99.2%. Regarding the chemical composition of this plant, the compounds usually found in *S. virgaurea* are saponins, which have been attributed to the ability to inhibit the transition from yeast to hyphal growth [98]. This attribution seems reasonable considering the inherent surfactant properties of saponins, as well as their iron chelator qualities, iron being necessary for the growth and development of *Candida* spp. [99].

**Table 1:** Medicinal plants with antimicrobial/antibiofilm activity against oral *Candida* spp. and the respective bioactive compounds present in their extracts.

Plant Name	Plant Extract	Compound	Microorganism	Results				References
				Antimicrobial Activity		Antibiofilm Activity		
*Allium sativum* L.	Essential oil (bulbs)	Allicin, alliin, ajoene [75]	*C. albicans* ATCC 14053	MIC	8.0 μg∙mL^−1^	>99.9% reduction	8.00 μg∙mL^−1^	[74]
				IZD	19.0 mm (50.0 μg∙mL^−1^)			
*Aloysia gratissima* (Aff & Hook) Tronc.	Essential oil (leaves)	*(E)*-pinocamphone, β-pinene, guaiol	*C. albicans* CBS 562	MIC	0.015 mg∙mL^−1^	12.3% inhibition	1.00 mg∙mL^−1^	[64]
				MFC	0.062 mg∙mL^−1^			
*Artemisia judaica* L.	Essential oil (aerial plant parts)	Piperitone, camphor, ethyl cinnamate, chrysanthenone	*C. albicans* ATCC 10231	MIC	1.25 μg∙mL^−1^	50.0% reduction	2.5 μg∙mL^−1^	[100]
*Brucea javanica* (L.) Merr.	Aqeuous extract (seeds)	Quassinoids, alkaloids,	*C. albicans* ATCC 14053	-		94.5% CSH reduction 79.7% adherence reduction	6.00 mg∙mL^−1^	[101]
			*C. dubliniensis* ATCC MYA-2975			90.4% CSH reduction 27.9% adherence reduction		
			*C. glabrata* ATCC 90030			84.8% CSH reduction 76.8% adherence reduction		
			*C. krusei* ATCC 14243			97.0% CSH reduction 67.6% adherence reduction		
			*C. lusitaniae* ATCC 64125			91.1% CSH reduction 89.0% adherence reduction		
			*C. parapsilosis* ATCC 22019			98.8% CSH reduction 49.0% adherence reduction		
			*C. tropicalis* ATCC 13803			88.4% CSH reduction 89.9% adherence reduction		
*Cassia spectabilis* DC.	Methanol extract (leaves)	(+)-spectaline; (−)-iso-6-cassine [102]	*C. albicans* 1 (CI)	MIC IZD	6.25 mg∙mL^−1^ 20 mm (100 mg∙mL^−1^)	97% inhibition	6.25 mg∙mL^−1^	[103]
			*C. albicans* 2 (CI)	MIC IZD	6.25 mg∙mL^−1^ 21 mm (100 mg∙mL^−1^)			
			*C. albicans* 3 (CI)	MIC IZD	6.25 mg∙mL^−1^ 23 mm (100 mg∙mL^−1^)			
*Chenopodium ambrosioides* L.	Aqueous extract (leaves)	Kaempferol, quercetin	*C. albicans* ATCC 90028	MIC	0.250 mg∙mL^−1^	>99.0% reduction	1.25 mg∙mL^−1^	[104]
				MFC	0.250 mg∙mL^−1^			
*Cinnamomum cassia* L. J.Presl	Essential oil (leaves, bark, stalk)	Cinnamaldehyde, benzyl benzoate, α-pinene	*C. albicans* ATCC 90028	MIC	65.5 µg∙mL^−1^	>99.9% reduction	1.00 mg∙mL^−1^	[80]
				MFC				
*Cinnamomum verum* J.Presl	Essential oil (leaves)	Eugenol, benzyl benzoate, *trans*-caryophyllene, acetyle eugenol, linalool	*C. albicans* ATCC MYA-2876	MIC	1.0 mg∙mL^−1^	50% reduction	0.15 mg∙mL^−1^	[105]
						50% inhibition	1.0 mg∙mL^−1^	
			*C. tropicalis* ATCC 750			50% reduction	0.35 mg∙mL^−1^	
						50% inhibition	>2.0 mg∙mL^−1^	
			*C. dubliniensis* ATCC MYA-646			50% reduction	0.2 mg∙mL^−1^	
						50% inhibition	0.2 mg∙mL^−1^	
*Cinnamomum zeylanicum* Blume	Essential oil (leaves)	Cinnamaldehyde, cinnamyl acetate, cinnamyl benzoate [79]	*C. albicans* ATCC 10231	MIC	0.1 µg∙mL^−1^	99.75% reduction	1.6 µg∙mL^−1^	[106]
				MFC	0.4 µg∙mL^−1^			
				IZD	42.5 mm (50 µg∙mL^−1^)			
*Coriandrum sativum* L.	Essential oil (leaves)	Decanal, *trans*-2-decenal, 2-decen-1-ol, cyclodecane, cis-2-dodecenal	*C. albicans* CBS 562	MIC	15.6 µg∙mL^−1^	53.43% inhibition	62.50 µg∙mL^−1^	[107]
				MFC	31.2 µg∙mL^−1^			
			*C. tropicalis* CBS 94	MIC	31.2 µg∙mL^−1^	89.76% inhibition	125 µg∙mL^−1^	
				MFC	62.5 µg∙mL^−1^			
			*C. krusei* CBS 573	MIC	15.6 µg∙mL^−1^	42.13% inhibition	15.62 µg∙mL^−1^	
				MFC	31.2 µg∙mL^−1^			
			*C. dubliniensis* CBS 7987	MIC	31.2 µg∙mL^−1^	61.51% inhibition	62.50 µg∙mL^−1^	
				MFC	62.5 µg∙mL^−1^			
			*C. rugosa* CBS 12	MIC	15.6 µg∙mL^−1^	68.03% inhibition	62.50 µg∙mL^−1^	
				MFC	31.2 µg∙mL^−1^			
*Croton urucurana* Baill.	Methanol extract (stems)	(epi)-catechin dimer I [108]	*C. albicans* ATCC 10231	-		46.0% inhibition	0.500 mg∙mL^−1^	[109]
*Cymbopogon citratus* (DC.) Stapf	Essential oil (leaves)	Citral, neral, β-myrcene, geraniol [110]	*C. albicans* ATCC 10231	MIC	0.1 µL∙mL^−1^	99.79% reduction	6.4 µL∙mL^−1^	[106]
				MFC	0.4 µL∙mL^−1^			
				IZD	18.2 mm (5% v.v^−1^)			
	Ethanol extract (leaves)	Citral, geraniol, neral, camphene, limonene [111]	*C. albicans* ATCC 18804	MIC	0.625 mg∙mL^−1^	>99.9% inhibition	3.13 mg∙mL^−1^	[112]
				MFC	2.50 mg∙mL^−1^	94.0% reduction	6.25 mg∙mL^−1^	
*Cymbopogon nardus* L. Rendle	Essential oil (leaves)	Citronellal, citronellol, geraniol	*C. albicans* ATCC 76645	MIC	32.0 µg∙mL^−1^	>99.0% inhibition	32.0 µg∙mL^−1^	[113]
				MFC				
*Cymbopogon winterianus* Jowitt	Essential oil (leaves)	Citronellal, citronellol, geraniol	*C. albicans* ATCC 90028	MIC	250 µg∙mL^−1^	>99.0% reduction	1.00 mg∙mL^−1^	[80]
				MFC				
*Cyperus articulatus* L.	Essential oil (bulbs)	α-pinene, mustakone, α-bulnesene	*C. albicans* CBS 562	MIC	0.125 mg∙mL^−1^	28.1% inhibition	1.00 mg∙mL^−1^	[112]
				MFC	0.500 mg∙mL^−1^			
*Eucalyptus globulus* Labill.	Essential oil (leaves)	Hyperoside, quercitrin, myricetin [114]	*C. albicans* ATCC 14053	MFC	0.219 mg∙mL^−1^	86% reduction	22.5 mg∙mL^−1^	[115]
			*C. tropicalis* ATCC 66029		0.885 mg∙mL^−1^	85% reduction		
			*C. glabrata* ATCC 66032		0.219 mg∙mL^−1^	85.2% reduction		
*Houttuynia cordata* Thunb	Ethanol extract (leaves)	Aldehydes	*C. albicans* CAD1	MFC	>2.17 mg∙mL^−1^	70.0% reduction	1.00% (*v/v*)	[116]
*Lippia sidoides* Cham.	Essential oil (leaves)	Thymol, *p*-cymene, α-caryophyllene	*C. albicans* CBS 562	MIC	0.250 mg∙mL^−1^	16.5% inhibition	1.00 mg∙mL^−1^	[117]
				MFC	0.500 mg∙mL^−1^			
*Melaleuca alternifolia* (Maiden & Betche) Cheel	Essential oil (leaves)	Terpinen-4-ol, *γ*-terpinene, *p*-cymene, *α*-terpinene,1,8-cineole, *α*-terpineol, *α*-pinene	*C. albicans* ATCC 18804	MIC	1.95 mg∙mL^−1^	MBEC	125 mg∙mL^−1^	[118]
	Essential oil (leaves)	Terpinen-4-ol, *γ*-terpinene, *α*-terpinene, terpinolene, 1,8-cineole	*C. albicans* ATCC 10231	MIC	3.40 mg∙mL^−1^	131% adherence reduction	0.75% (*v/v*)	[119]
			*C. albicans* SC5314	MIC	0.84 mg∙mL^−1^	76.0% adherence reduction		
*Mikania glomerata* Spreng	Essential oil (leaves)	Germacrene D, α-caryophyllene, bicyclogermacrene	*C. albicans* CBS 562	MIC	0.250 mg∙mL^−1^	22.7% inhibition	1.00 mg∙mL^−1^	[117]
				MFC	0.250 mg∙mL^−1^			
*Piper betle* L.	Aqueous extract (leaves)	Hydroxychavicol, cinnamoyl derivatives, luteolin, apigenin [120]	*C. albicans* ATCC 14053	-		38.6% CSH reduction 61.4% adherence reduction	6.00 mg∙mL^−1^	[101]
			*C. dubliniensis* ATCC MYA-2975			78.3% CSH reduction 21.4% adherence reduction		
			*C. glabrata* ATCC 90030			71.4% CSH reduction 12.4% adherence reduction		
			*C. krusei* ATCC 14243			31.6% CSH reduction 56.4% adherence reduction		
			*C. lusitaniae* ATCC 64125			67.5% CSH reduction 47.6% adherence reduction		
			*C. parapsilosis* ATCC 22019			48.1% CSH reduction 46.5% adherence reduction		
			*C. tropicalis* ATCC 13803			29.7% CSH reduction 86.9% adherence reduction		
*Rosmarinus officinalis* L.	Liposoluble extract (leaves)	Carnosic acid, carnosol [121]	*C. albicans* ATCC 18804	MIC	0.78 mg∙mL^−1^	99.9% reduction	200 mg∙mL^−1^	[122]
				MMC	3.13 mg∙mL^−1^			
*Satureja hortensis* L.	Essential oil (leaves and flowers)	Thymol, λ-terpinene, carvacrol, *p*-cymene	*C. albicans* F81 (CI)	MIC MFC	300 µg∙mL^−1^ 400 µg∙mL^−1^	91.0% inhibition 91.0% reduction	4.80 mg∙mL^−1^	[123]
			*C. albicans* F94 (CI)		200 µg∙mL^−1^ 300 µg∙mL^−1^	90.0% inhibition 80.0% reduction		
			*C. albicans* F87 (CI)		300 µg∙mL^−1^ 400 µg∙mL^−1^	86.0% inhibition 76.0% reduction		
			*C. albicans* F49 (CI)		400 µg∙mL^−1^ 600 µg∙mL^−1^	92.0% inhibition 92.0% reduction		
			*C. albicans* F82 (CI)		400 µg∙mL^−1^ 600 µg∙mL^−1^	89.0% inhibition 89.0% reduction		
			*C. albicans* F95 (CI)		400 µg∙mL^−1^	81.0% inhibition 81.0% reduction		
			*C. albicans* F92 (CI)		300 µg∙mL^−1^ 600 µg∙mL^−1^	90.0% inhibition 90.0% reduction		
			*C. albicans* F60 (CI)		400 µg∙mL^−1^ 600 µg∙mL^−1^	80.0% inhibition 80.0% reduction		
			*C. albicans* F86 (CI)		200 µg∙mL^−1^ 300 µg∙mL^−1^	87.0% inhibition 87.0% reduction		
			*C. albicans* F91 (CI)		300 µg∙mL^−1^ 400 µg∙mL^−1^	83.0% inhibition 83.0% reduction		
			*C. albicans* F69 (CI)		200 µg∙mL^−1^ 300 µg∙mL^−1^	91.0% inhibition 80.0% reduction		
			*C. albicans* F1 (CI)			87.0% inhibition 79.0% reduction		
			*C. albicans* F34 (CI)			86.0% inhibition 91.0% reduction		
			*C. albicans* F19 (CI)			90.0% inhibition 85.0% reduction		
			*C. albicans* F78 (CI)		400 µg∙mL^−1^ 600 µg∙mL^−1^	84.0% inhibition 84.0% reduction		
*Schinus terebinthifolia* Raddi.	Methanol extract (leaves)	Phenolic compounds, anthraquinones, terpenoids, alkaloids	*C. albicans* ATCC 10231	-		47.0% inhibition	0.007 mg∙mL^−1^	[109]
*Solidago virgaurea* subsp. *alpestris* Waldst. & Kit. ex Willd.	Aqueous extract (aerial plant parts)	Saponins	*C. albicans* ATCC 10231	NA (IZD)		95.9% inhibition 92.4% reduction	0.250 mg∙mL^−1^ 0.750 mg∙mL^−1^	[98]
			*C. albicans* IM001 (CI)			96.0% inhibition 82.2% reduction	0.250 mg∙mL^−1^ 0.750 mg∙mL^−1^	
			*C. albicans* IM003 (CI)			99.5% inhibition 76.3% reduction	0.250 mg∙mL^−1^ 0.750 mg∙mL^−1^	
			*C. albicans* IM007 (CI)			95.1% inhibition 91.9% reduction	0.250 mg∙mL^−1^ 0.750 mg∙mL^−1^	
*Solidago virgaurea* L. subsp. *virgaurea*.	Aqueous extract (aerial plant parts)	Saponins	*C. albicans* ATCC 10231	NA (IZD)		98.4% inhibition 77.9% reduction	0.250 mg∙mL^−1^ 0.750 mg∙mL^−1^	
			*C. albicans* IM001 (CI)			99.2% inhibition 91.1% reduction	0.250 mg∙mL^−1^ 0.750 mg∙mL^−1^	
			*C. albicans* IM003 (CI)			97.3% inhibition 79.2% reduction	0.250 mg∙mL^−1^ 0.750 mg∙mL^−1^	
			*C. albicans* IM007 (CI)			96.5% inhibition 90.9% reduction	0.250 mg∙mL^−1^ 0.750 mg∙mL^−1^	
*Terminalia catappa* L.	Ethanol extract (leaves)	Caffeic acid, quercitrin, kaempferol, gallic acid, chlorogenic acid, isoquercitrin [124]	*C. albicans* ATCC 90028	MIC MFC	6.25 mg∙mL^−1^ 12.5 mg∙mL^−1^	>98.0% reduction	62.5 mg∙mL^−1^	[125]
	*n*-butanol fraction from ethanol extract (leaves)		*C. albicans* ATCC 90028	MIC MFC	250 μg∙mL^−1^	>99.5% reduction	2.50 mg∙mL^−1^	[126]
			*C. glabrata* ATCC 2001	MIC MFC	250 μg∙mL^−1^	>99.0% reduction	2.50 mg∙mL^−1^	
*Trachyspermum ammi* (L.) Sprague	Aromatic water (aerial plant parts)	Thymol, carvacrol, carvotanacetone	*C. albicans* CBS1905	-	-	95.2% inhibition	0.5% (*v*/*v*)	[127]
*Zataria multiflora* Boiss.	Aqueous extract (whole plant)	Thymol, hydroxyl benzoic acid, and cymene [128]	*C. albicans* PTCC-5027	MIC	1.50 mg∙mL^−1^	87% reduction	25 mg∙mL^−1^	[129]
	Ethanolic extract (whole plant)			MIC	0.84 mg∙mL^−1^	97% reduction		

^1^ IZD: Inhibition zone diameter; MIC: Minimum inhibitory concentration; MFC: minimum fungicidal concentration; MMC: minimum microbiocidal concentration; MBIC: Minimum biofilm inhibitory concentration; MBEC: Minimum biofilm eradication concentration; NA: No activity; -: Not tested; CI: clinical isolate; CSH: Cell surface hydrophobicity.

## 5. Conclusions

Medicinal plants are still an untapped source of powerful natural products with great antimicrobial and/or antibiofilm potential, especially in a backdrop of increasing antibiotic resistance. This review aimed to identify medicinal plant products, such as essential oils and plant extracts for the treatment of common oral *Candida* infections, mainly caused by the formation of fungal biofilms. Although extracts from many medicinal plants have shown exciting results in controlling these biofilms, the most promising plant extracts were from *A. sativum* L., which reduced *C. albicans* ATCC 14053 oral biofilm formation by more than 99.9% at a concentration of 8.0 µg∙mL^−1^; the essential oil extracted from *C. zeylanicum* Blume., which showed, at a concentration of 1.6 µg∙mL^−1^, a reduction in oral biofilm formation by *C. albicans* ATCC 10231 higher than 99.75%; and the essential oil obtained from *C. citratus* (DC) Stapf, which exhibited a reduction in the oral biofilm formation by *C. albicans* ATCC 10231 greater than 99.79% at 6.4 µg∙mL^−1^. Interestingly, in all of these medicinal plant species, organic compounds with proven bioactive properties such as antimicrobial and antibiofilm effects were identified.

The use of essential oils and plant extracts from medicinal plants can be a great alternative to conventional antimicrobials in the treatment of fungal infections in the oral cavity since they have low levels of cytotoxicity and, to date and to our knowledge, do not induce resistance in microorganisms. However, research on the use of medicinal plants in the treatment of oral ailments remains an extremely interesting and unexplored topic, mainly due to the wide variety of plants whose phytochemical profiles are still unknown, and which will likely show good antimicrobial and antibiofilm properties.
